# Epidemic evolution models to the test of Covid-19

**DOI:** 10.1007/s40574-020-00252-7

**Published:** 2020-08-03

**Authors:** Primo Brandi, Rita Ceppitelli, Anna Salvadori

**Affiliations:** Department of Mathematics and Computer Sciences, University of Perugia, Via L. Vanvitelli 1, 06123 Perugia, Italy

**Keywords:** Mathematical models, Epidemic evolution dynamic, Iterative process, Fitting data, 92D30, 65D05, 68N01

## Abstract

We illustrate a suitable adaptation and modification of classical epidemic evolution models that proves helpful in the study of Covid-19 spread in Italy.

## Introduction

Covid-19 pandemic has brought the importance of mathematical modeling to predict phenomena, even in the presence of great uncertainty, before the eyes of the whole society.

From the beginning the virologists have been very cautious, declaring that they were faced with a new virus whose behavior they ignored. On the base of the experience gained with other epidemics, they could only recommend an immediate lockdown to try to reduce the damage.

Politicians were in serious difficulty to make such drastic decisions that could have had serious economic and social consequences.

At this point the mathematicians entered the field and the forecast based on mathematical models became the landmark to which everyone clung. Thus the media were filled with colorful histograms and graphics which everyone looked at as foolproof oracles. The language of modeling entered by force into the everyday language: parameters, trends, peaks, inflection points, asymptotic behavior etc.

However, in our view a “collateral problem” arises, which has perhaps not been given much importance. The mathematical language, even if popularized, involves precise terms and rigorous rules that cannot be derogated, under penalty of canceling their effectiveness.

We do believe that it is necessary to update the basic culture of the average citizen, with some elements of this language, in order to avoid dangerous misunderstandings. In the front line, the School is called to this mission, but all the researches should contribute.The present article, written in this spirit, aims to present some elementary models on epidemics spread and to discuss their possible use in the study of Covid-19 in Italy. As it will be discussed in details, classical SIR model [3–5], which is very effective for describing seasonal flu, is not adequate for Covid-19 epidemic. The essential reason is that the two main parameters that control the degree of infectiveness and that of healing are assumed constant. This is not true at all in Covid-19 also because the containment measures have strongly interacted with the epidemic spread. For this reason we introduce a suitable modification of the point of view that produces an adaptated SIR* model, useful and effective to describe Covid-19 spread.In this way, we am to spread the spirit of mathematical modeling and also to highlight some fundamental attribute together with critical aspects.

Numerical data processing and graphic representations were carried out using Wolfram’s Mathematica 11.

## Elementary epidemic evolution models

The simplest epidemic models assume that the population under observation is *isolated* and does not change during the epidemic. This implies that the number of individuals is constant, in particular, neither births nor deaths are considered, except those possibly due to the epidemic.

More precisely, the population is divided into three categories:

- the *infectious* (I): subjects carrying the disease and able to transmit it;
- the *susceptible* (S): not yet sick subjects, that could contract the disease;
- the *removed* (R): subjects who have been sick, but who are no longer; they who cannot infect other individuals or get sick again (healed, immunized or dead).

Let’s start with a very simplified model that takes into account only infectious and susceptible.

### SI Model

The model describes the evolution of an infectious disease which under the following assumptions:

- there are not removed;
- the transition from infectious to susceptible is not possible;
- the contagion occurs by direct contact, in particular it is assumed that the incidence of the disease (the infectious rate) is proportional to both the number of infectious and that of the susceptibles (i.e. the subjects who are not yet infected).

If *P* is the number of the whole population, denoted by $I_n$ and $S_n$ respectively the number of the subjects of the two categories present at the step *n*, we have $I_n + S_n = P.$ The situation is therefore described by the iterative process

$$\begin{aligned} (SI) \ \left\{ \begin{array}{l} I_0 \ \ \text{ start }\\ I_{n+1}-I_n = \beta I_nS_n = \beta I_n(P-I_n), \quad n=0,1,2,\ldots \end{array} \right. \end{aligned}$$

where $\beta$ is called *virulence factor*.

With obvious algebraic manipulations, we get it

$$\begin{aligned} (SI) \ \left\{ \begin{array}{l} I_0 \ \ \text{ start }\\ I_{n+1} = (\beta P+1)I_n - \beta I_n^2, \quad n=0,1,2,\ldots \end{array} \right. \end{aligned}$$

**Fig. 1:**
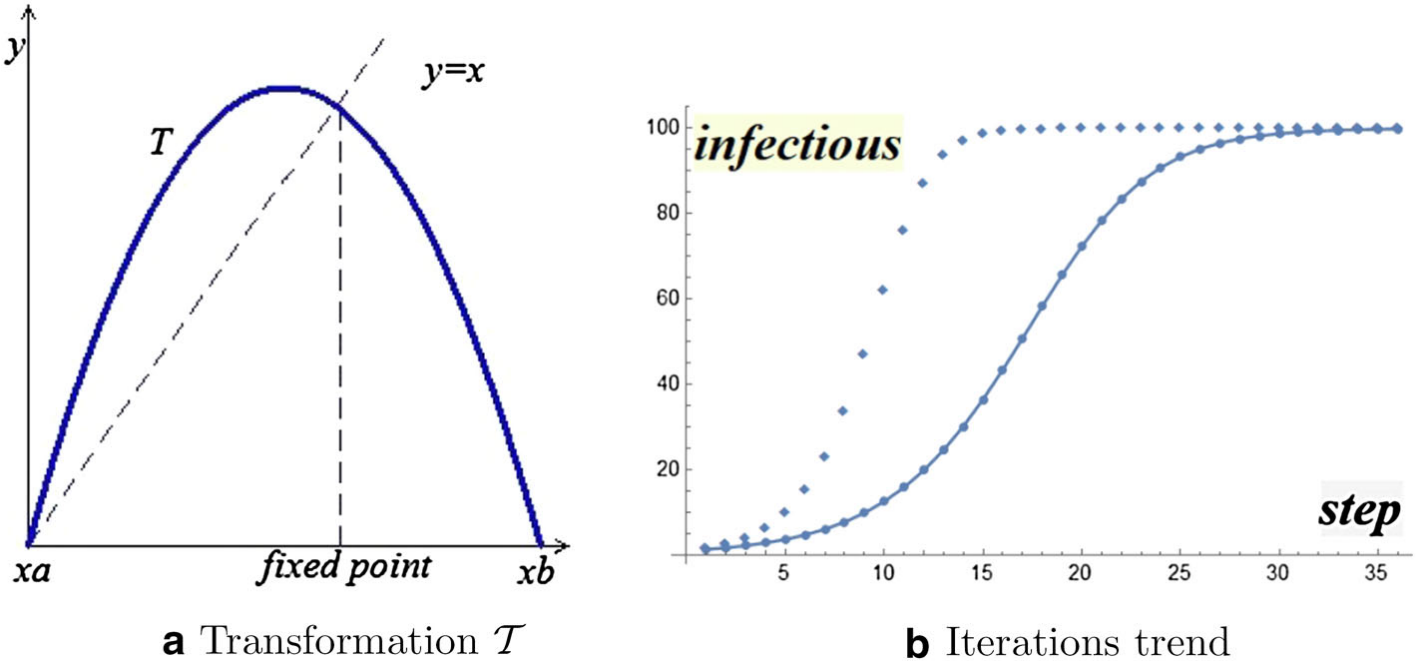
Infectious evolution

This shows that the process is governed by the transformation (Fig. 1a)

$$\begin{aligned} T(x) = - \beta x^2 + (\beta P+1)x \end{aligned}$$

as it occurs for the well-known non-linear model on population evolution due to Verhulst [1, Cap. 2], [2].

As it is well known, the process does not admit a closed formula, we therefore resort to numerical simulations.

Figure 1b shows the iterations trends for a population $P=100$ with initial incidence $I_0=1$, under different virulence factors $\beta =0.003 \ \ \beta =0.006$

As we can see, a logistic trend holds in both cases; the asymptotic evolution foresees a pandemic, where the whole population is infected over time. The virulence factor $\beta$ does not affect the final asymptotic evolution, but only the infectious rate of growth.

Since SI model can reveal unrealistic, we take a look at its variant SIS.

### SIS Model

SIS *model provides the possibility of healing, even if the healed subjects do not acquire immunity and return to be susceptible again. Precisely it is assumed that the cure rate is proportional to the number of infectious.*

The formulation of SIS model is the following extension of SI one

$$\begin{aligned} (SIS) \ \ \left\{ \begin{array}{l} I_0 \ \ \text{ start }\\ I_{n+1}-I_n = \beta I_n(P-I_n) - \gamma I_n, \quad n=0,1,2,\ldots \end{array} \right. \end{aligned}$$

where $0<\beta <1$ is the *virulence factor* and $0<\gamma <1$ it is the *healing factor*.

SIS model is written equivalently

$$\begin{aligned} (SIS)\ \ \left\{ \begin{array}{l} I_0 \ \ \text{ start }\\ I_{n+1}-I_n = (\beta P-\gamma )I_n - \beta I_n^2, \quad n=0,1,2,\ldots \end{array} \right. \end{aligned}$$

The process is governed by a quadratic transformation

$$\begin{aligned} T(x) = - \beta x^2 + (\beta P-\gamma +1)x \end{aligned}$$

similar to SI model, but where the presence of the parameter $\gamma$ plays a determining role, as we will see.

Let’s study the main characteristics of the transformation *T* on the domain $E=[0, \frac{1+\beta P-\gamma }{\beta }]$.

Assumed the constraint $\ T(E) \subset E$ and put $k = 1+\beta P-\gamma$ , let us consider the following two cases which are the only interesting ones from the epidemiological point of view.

**Fig. 2:**
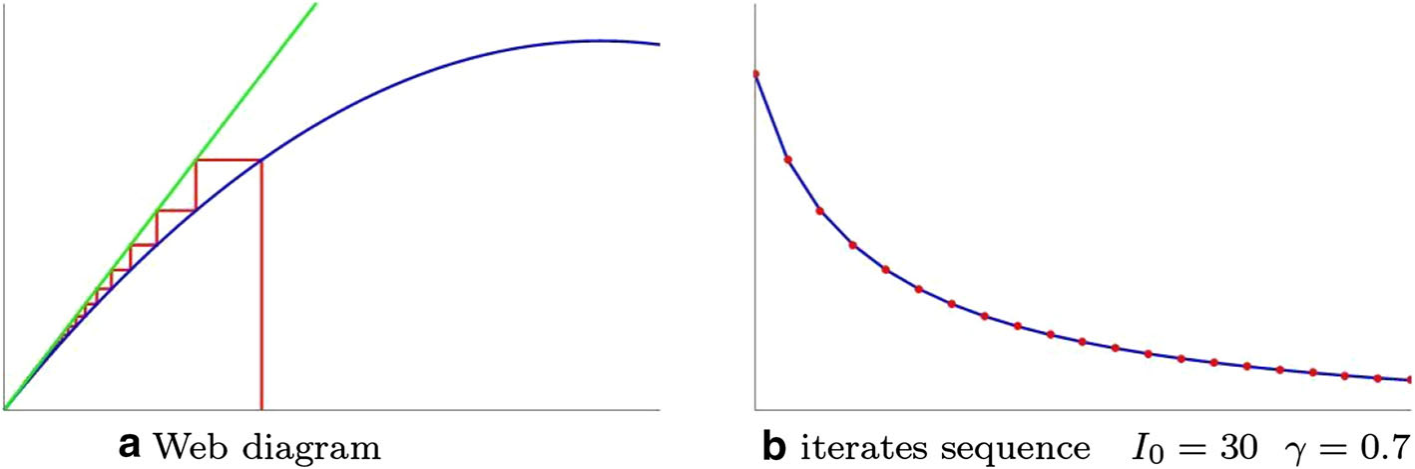
**a** Web diagram **b** iterates sequence

**First case: epidemic disappearance** (Fig. 2a, b) In the case $0<k\le 1$ transformation *T* admits the single fixed point $\bar{x}=0$.Whatever the start situation, the phenomenon evolves towards the complete extinction of infectious, with the disappearance of the epidemic.

**Fig. 3:**
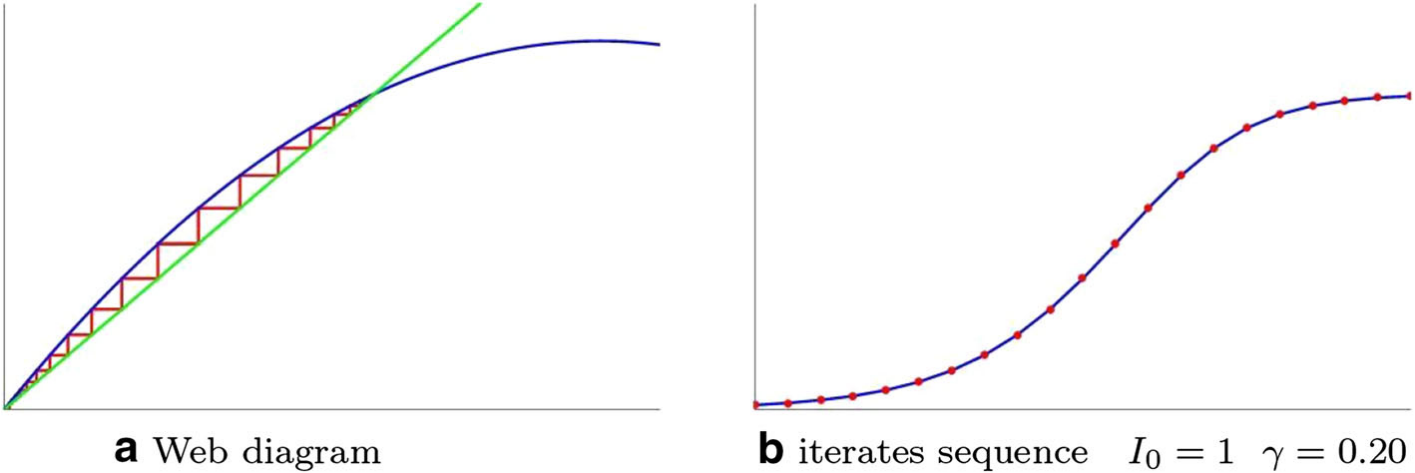
**a** Web diagram **b** iterates sequence

**Second case: endemic balance ** (Fig. 3a,b) If $1<k\le 2$, the transformation admits two fixed points, but only one is an attractor. Whatever the start, the number of infectives grows (logistically) towards the fixed point $\bar{\bar{x}} = P -\frac{\gamma }{\beta }$. The situation evolves towards an endemic balance in which susceptible and infectious coexist.

The examples show, as predictable, that the healing factor plays a fundamental role in the evolution of the phenomenon. With the same population $P=100$ and infectivity factor $\beta =0.0065$, we get the following different evolutions

- for small values of $\gamma$ the situation evolves towards an endemia (Fig. 3b);
- for sufficiently high values of the healing factor, the epidemic ends (Fig. 2b). Precisely the threshold value between endemia and epidemic extinction is
  $$\begin{aligned} \beta P-\gamma = 0 \ \Leftrightarrow \ \frac{\gamma }{\beta } = P \end{aligned}$$
  a balance of virulence and healing factors.

Both SI and SIS models are not suitable for the description of seasonal flu evolution as the curve of infectives lacks the “classic peak” present in the flu charts [6].

In 1927 the biochemists William Kermack and Anderson McKendrick [3–5] proposed a model (known as SIR) for the study of an epidemic that is well suited to the flu case. The new model is more complex than those already seen since it involves all three types of subjects.

### SIR Model

SIR *model* (*Susceptible, Infectious, Removed*) *describes the evolution of a curable infectious disease, which confers immunity* (*at least temporary*). *Contagion occurs by direct contact.*

Let’s denote by $I_n, S_n$ and $R_n$ respectively the number of the subjects of the three categories (infectious, susceptible and removed) present at the step n. Recalling that the population is isolated, it turns out $I_n+S_n+R_n=P.$ As the infection spreads, some individuals move from susceptible to infectious. The number of infectious changes for two reasons: new people get sick and some sick people pass removed (recovered or dead). In other words

$$\begin{aligned} I_{n+1}-I_n = -(S_{n+1}-S_n) - (R_{n+1}-R_n) \end{aligned}$$

Kermack and McKendrick formulate the following assumptions.

**Principle of susceptible variation** The susceptible rate is proportional to the number of contacts between susceptible and infectious:

$S_{n+1}-S_n = -\beta S_nI_n$ where the constant $\beta$ is called *virulence factor.*

**Principle of removed variation** The removed rate is proportional to the number of infectious:

$R_{n+1}-R_n = \gamma I_n$ where the constant $\gamma$ is called *healing factor.*

Thus the infectious rate is

$$\begin{aligned} I_{n+1}-I_n = \beta S_nI_n-\gamma I_n \end{aligned}$$

Finally, we obtain the following iterative process

$$\begin{aligned} (SIR) \ \left\{ \begin{array}{l} I_0, S_0, R_0 \ \ \text{ start }\\ S_{n+1}-S_n = -\beta S_nI_n\\ I_{n+1}-I_n = \beta S_nI_n-\gamma I_n\\ R_{n+1}-R_n = \gamma I_n \quad n=0,1,2,\ldots \end{array} \right. \end{aligned}$$

subjected to the constraint $I_n+S_n+R_n=P$

It is easy to prove that the process is governed by the non-linear vector transformation $T=(T_1,T_2): \mathbb {R}^2 \rightarrow \mathbb {R}^2$

$$\begin{aligned} \ \left\{ \begin{array}{l} T_1(x,y) = x - \beta xy\\ T_2(x,y) = (1-\gamma )y + \beta xy\\ \end{array} \right. \end{aligned}$$

The two-dimensional, non-linear model is more complex than those already seen. Let’s test SIR model in the case of flu epidemic.

**Flu epidemic in Italy. Seasons 2017–2018 and 2018–2019.**

**Fig. 4:**
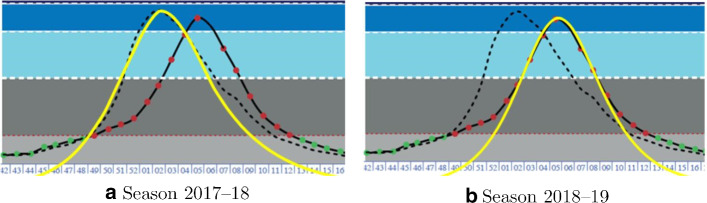
Flu epidemic on Italy

Figure 4a, b show the real data trend of the flu in Italy over the past two years (dashed and dark curves)[6].

A numerical simulation of the flu epidemic in 2017–2018, obtained in force of SIR model, produces the clear curve in Fig. 4a. As can be seen, the model significantly fits the evolution in the central 10 weeks (from the 49th of 2017 to the 7th of 2018), when the “pea” occurs.

The simulation relating to the season 2018–2019 confirms a similar agreement between SIR model and the real data around the “peak” (Fig. 4b).

## Covid-19 epidemic in Italy

In light of the examples of the flu epidemic, we can ask ourselves:

*is the SIR model suitable for describing the current Covid-19 epidemic?*

Let’s analyze the situation. The data made available by the Protezione Civile [7] allow to know, day after day the number of infectious $I_n$ and that of removed $R_n$. Of course, being an epidemic due to a new virus, we do not know either the *virulence factor* $\beta$ or the *healing factor* $\gamma$. It is also difficult to estimate the number of susceptible. Assuming *P* equal to the entire Italian population (60 million individuals) is not meaningful. Apparently the issue seems like an “impossible mission”. We will presents our analysis of the situation in three key moments.

**First forecast. After 4 weeks, before the effects of containment measures**.

**Fig. 5:**
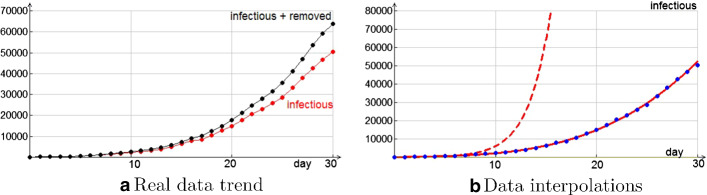
Epidemic evolution—first month

The first useful thing to do, as mathematical modeling teaches, is to study data trends. Figure 5a describes the epidemic growth in the month under observation: it shows a fast growth that has just started to slow down. One could conjecture an exponential growth for both the sequences, as many media affirmed. It is easy to check that this is not the case, we must therefore proceed with a more accurate analysis. Figure 5b clearly shows the real growth trend of infectious.

The initial exponential growth lasted about 1 week (dashed line), then it has become only cubic (continuous line). Note that if the growth had remained exponential, the current number of infected would have been reached in just fifteen days! Evidently the partial containment measures that have been adopted have had an important effect.

If we want to make the analysis on the epidemic evolution meaningful, a mathematical modelling is necessary. In light of what has been seen for the flu epidemic, we could try to adopt SIR model.

We recall that the two main terms of the model are

- the healing term $\gamma I_n$, where the constant $\gamma$ is the *healing parameter*;
- the infectivity term $\beta S_nI_n$ which takes into account the number of *contacts* between susceptible and infected, where the constant $\beta$ is the *infectivity parameter*.

Unfortunately, in the present situation the estimation of both the parameters presents some problems.

**Fig. 6:**
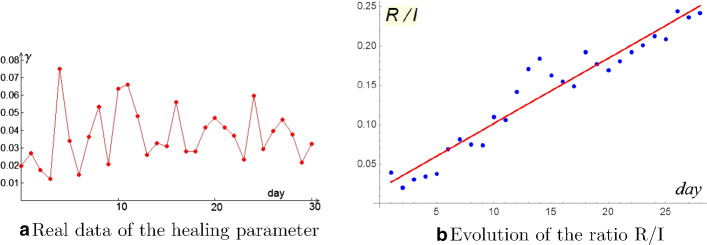
Discussion of the healing term

Let’s start with the healing parameter $\gamma = \frac{R_{n+1}- R_n}{I_n}$ which is not constant at all, but it highly depends on time (Fig. 6a). Moreover it doesn’t look productive to replace the parameter $\gamma$ by a sequence $\gamma _n$ obtained by means of interpolation techniques. Therefore we must necessarily change the point of view. An idea could be to analyze how the ratio between removed and infected varies over time $\frac{R_n}{I_n} = g_n$ (Fig. 6b).

Figure 6b suggests to adopt a linear regression

$$\begin{aligned} g(t)=0.0083t+0.019\quad R^2=0.9331 \end{aligned}$$

As a consequence we obtain

$$\begin{aligned} R_{n+1}- R_n = g(n+1)I_{n+1}-g(n)I_n \end{aligned}$$

This equation replace the third one in SIR model.

About the infectivity term $\beta S_n I_n$, as already observed we don’t have susceptible data available. An idea could be to give up estimating the infectivity parameter and discuss the behavior of the whole product

$$\begin{aligned} \beta S_n = \frac{I_{n+1}- I_n + R_{n+1}- R_n}{I_n} \end{aligned}$$

**Fig. 7:**
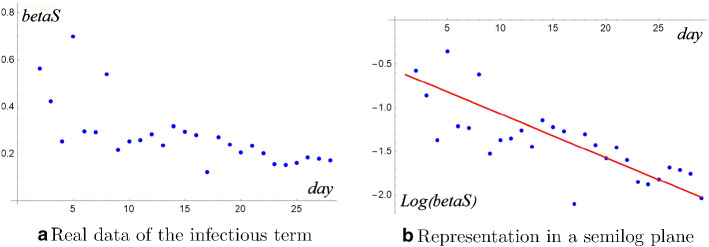
Discussion of the infectious term

Figure 7a shows that sequence $\beta S_n$ is highly depending on time variable. Of course a linear regression is not adequate since a close possibility of zero “contact” is not reasonable.

In order to choose a suitable fitting, let us passing to a semilog plane where the linear regression fits very well (Fig. 7b).

$$\begin{aligned} \log [\beta S(t)]=-0.052 t-0.55 \quad R^2=0.9704 \end{aligned}$$

Therefore the right regression is of exponential type $\beta S(t)=Be^{-kt}.$

Finally, we obtain the following iterative process, that can be seen as a variant of the classic SIR model

$$\begin{aligned} (SIR^*) \ \left\{ \begin{array}{l} I_0, R_0 \ \ \text{ start }\\ R_{n+1}- R_n = g(n+1)I_{n+1}-g(n)I_n\\ I_{n+1}-I_n= \beta S(n)I_n-[g(n+1)I_{n+1}-g(n)I_n] \quad n=0,1,2,\ldots \end{array} \right. \end{aligned}$$

At a first glance it could appear an implicit iterative process; but an easy algebraic manipulation leads to the following formulation

$$\begin{aligned} (SIR^*) \ \left\{ \begin{array}{l} I_0, R_0 \ \ \text{ start }\\ I_{n+1}=I_n\frac{1+g(n)+\beta S(n)}{1+g(n+1)} \\ R_{n+1}= R_n + g(n+1)I_{n+1}-g(n)I_n \quad n=0,1,2,\ldots \end{array} \right. \end{aligned}$$

SIR* model produces forecast F1 in Fig. 9a. A very alarming prediction where the peak would be reached only at the 12th week with an infected level more than triple of the present one $I_{28}$. The descent would be extremely slow. Furthermore, the total number of infected and removed would exceed 250 thousand (Fig. 9b). Fortunately this did not occur, but only thanks to the containment measures probably adopted on the basis of similar assessments.

**Second forecast. After 8 weeks, in full lockdown**.

**Fig. 8:**
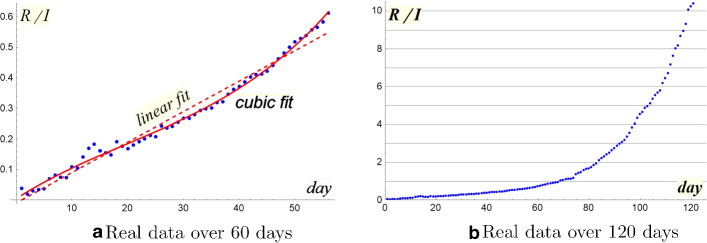
Evolution of the ratio R/I

We modify our forecast, on the basis of a suitable updating the two basic interpolating functions *g* and $\beta S$. Note that a linear regression does not fit the new trend for ratio $\frac{R_n}{I_n}$ as Fig. 8a shows. Since the growth appears superlinear, at least for the values of the second month, we can adopt a cubic interpolation, which reveals to be very operative. Indeed, we have:

$$\begin{aligned}&\hbox {linear interpolation: } R^2=0.9751\\&\hbox {cubic interpolation } R^2_{adj}=0.9931 \end{aligned}$$

**Fig. 9:**
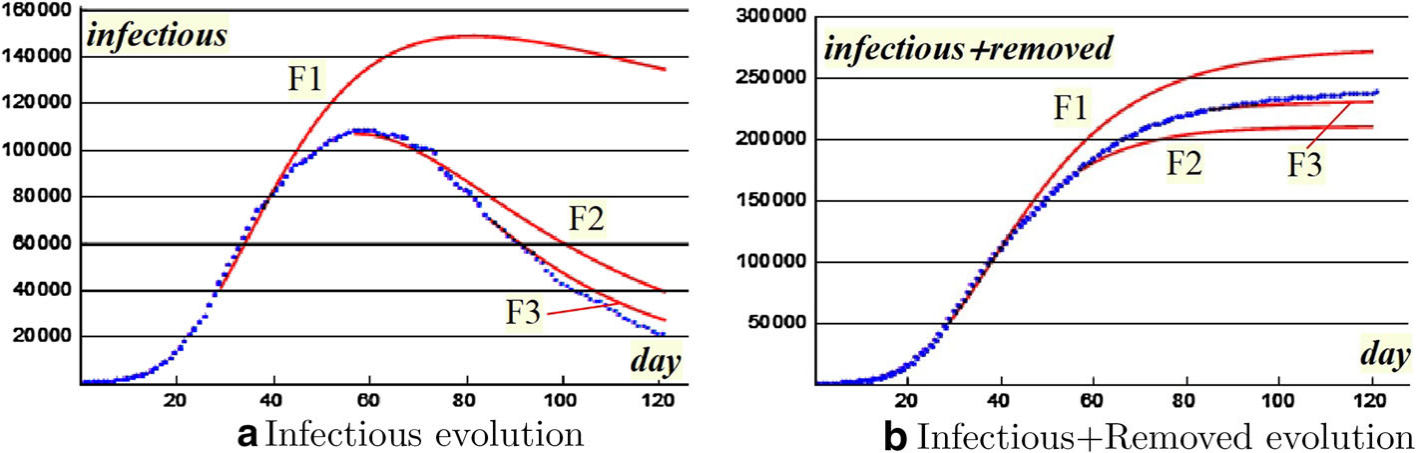
Forecasts according to SIR* model

For the sequence $\beta S_n$  an exponential regression looks still suitable (see Fig. 9b in semilog plane), we have just to update the parameters:

$$\begin{aligned} \log \beta S(t)=-0.056t-0.5\quad R^2=0.9669 \end{aligned}$$

SIR* model produces the forecast F2 which looks much better than the previous one. The peak has just been achieved, with a maximum of about 110,000 infectious. F2 in Fig. 9a follows the peak very well (either in time or in volume) even if the expected descent curve is over estimated. The total number of involved subjects would be lower than the previous forecast: just above 200,000 (Fig. 9b). This significant improvement demonstrates the validity of the confinement, but the slow decline of infectious still suggests caution.

**Third forecast. Immediately after the end of lockdown (after 12 weeks)**.

Let’s evaluate the positive effects of the confinement measures. As perhaps it was natural to expect, the choice of fitting still works well; we have just to update the parameters:

*g* cubic interpolation $R^2_{adj}=0.9943$; $\log [\beta S]$ linear regression $R^2=0.9581$.

As we can see in Fig. 9a, b, forecasts F2 and F3 follow the epidemic curve better and better. The excellent result lies on the choice of the cubic interpolation that chases the strong growth of the ratio $$R_n / I_n $$ (Fig. 8a). As Fig. 8b shows, the removed growth has significantly increased after the first two months, thanks to the large number of healed.

**Fig. 10:**
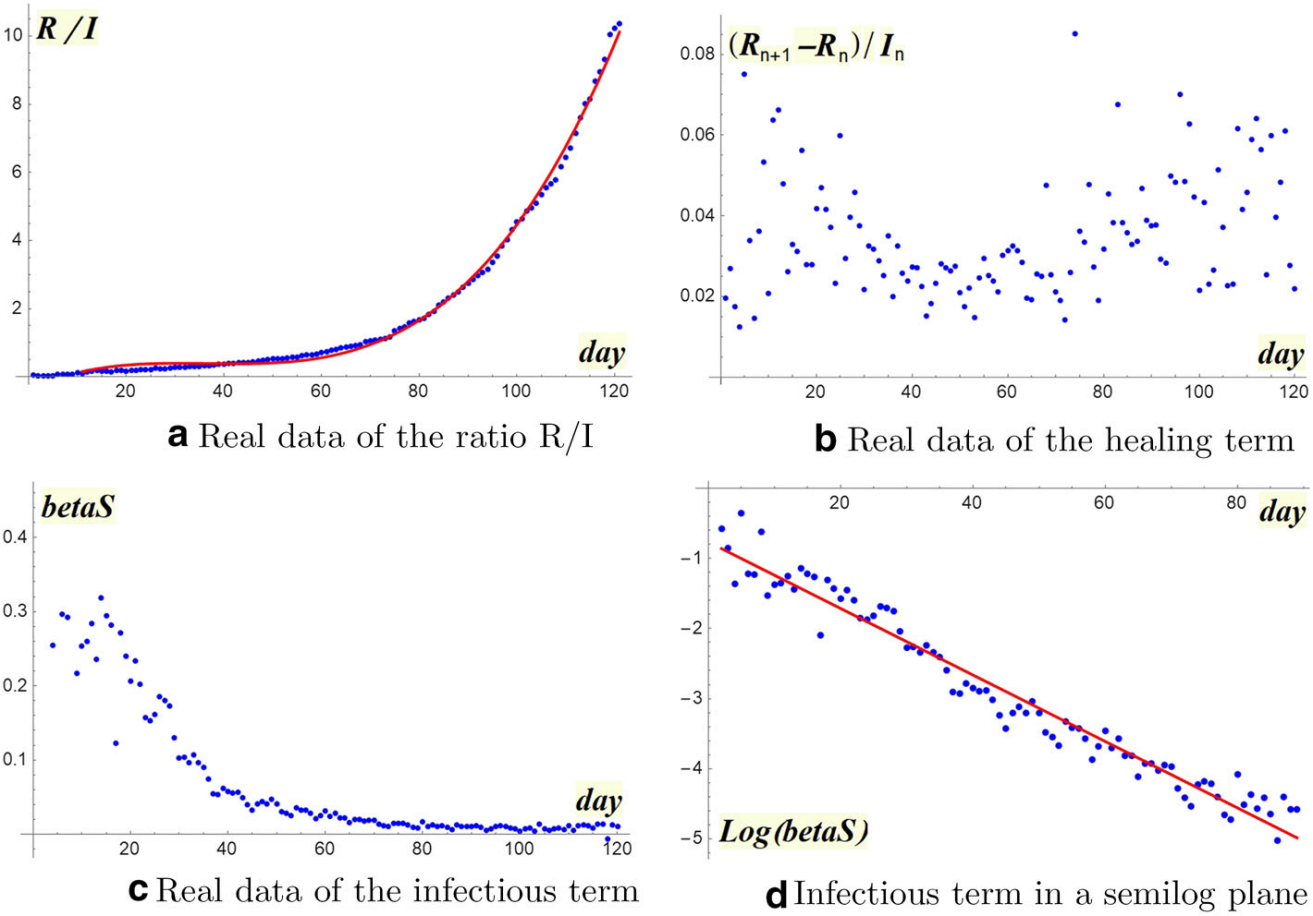
Discussion of real data over 120 days

Now that we have epidemic data of over 120 days (22.2–26.6.2020), we can try a backward evaluation of SIR* model. The images (Fig. 10a–d) confirm the goodness of our new approach: as can be clearly seen, the ratio $R_n /I_n$ remained *regular* throughout the period, while the infectivity factor $(R_{n+1}-R_n)/I_n$ continued to fluctuate highly. Also the term $\beta S$ maintained a strong exponential regularity.

## Conclusions

The good performance of forecasts F2 and F3 could lead to believe that we have accomplished our *mission impossible*. Actually, we need to be more cautious.

Of course, on the popularize side, we have half-opened the doors of modeling workshops to outsiders; while on the scientific size, we have done only the first steps. Our modification of SIR model consists essentially in changing the point of reference: a system on initial conditions has become a process that takes into account the historical data strongly. This aspect probably deserves further analysis. For example, the effectiveness of SIR* model on Covid-19 epidemic could be assessed in other Countries. Moreover it would be interesting to tackle a SIR* model of continuous type. In conclusion, the mission continues! As often happens in mathematical research, the solution of a problem opens another hundred; perhaps this is great part of its charm.
